# Why Most Child Sexual Abuse Examinations Are Normal: Forensic Implications, Misconceptions, and Evidence-Based Communication

**DOI:** 10.7759/cureus.105942

**Published:** 2026-03-26

**Authors:** Abdulkreem Al-Juhani, Naif Aljohani, Fatimah R Al Hejji, Norah S Alibrahim, Mehad F Abdelgalil, Maryam Y Alabdullah, Khalid H Alzahrani

**Affiliations:** 1 Forensic Medicine, Forensic Medicine Center, Jeddah, SAU; 2 Surgery, Faculty of Medicine, King Abdulaziz University, Jeddah, SAU; 3 Forensic Medicine, Riyadh Forensic Medicine Center, Riyadh, SAU; 4 College of Medicine, Almaarefa University, Diriyah, SAU; 5 Familly Medicine, King Faisal University, Al-Ahsa, SAU; 6 Paediatrics, King Faisal University, Al-Ahsa, SAU; 7 Paediatrics, University of Khartoum, Khartoum, SDN; 8 Paediatrics, Mansoura University, Mansoura, EGY; 9 Medicine, Faculty of Medicine, King Abdulaziz University, Jeddah, SAU

**Keywords:** anogenital findings, child sexual abuse, hymen, pediatric forensic examination, photodocumentation, sti testing, testimony

## Abstract

Child sexual abuse (CSA) evaluations frequently yield normal or nonspecific anogenital findings, even in cases later substantiated through investigation. This reality is often misunderstood in clinical, legal, and multidisciplinary contexts, where the absence of physical injury may be incorrectly interpreted as the absence of abuse. The purpose of this narrative review is to explain why most CSA examinations are normal and to provide an evidence-based framework for interpreting and communicating these findings in a manner that is medically accurate and forensically defensible. A targeted review of contemporary guidelines, large clinical series, healing studies, forensic evidence collection literature, and sexually transmitted infection (STI) testing recommendations was conducted. Evidence consistently demonstrates that low physical yield is driven by several converging factors: the nature of abusive acts, the elasticity and rapid healing capacity of pediatric anogenital tissues, delayed disclosure leading to non-acute examinations, variability in examiner experience and documentation quality, and the time-sensitive nature of biological evidence recovery. STI findings are uncommon overall and require contextual interpretation, including consideration of testing methods and potential nonsexual transmission pathways. Importantly, a normal examination neither confirms nor excludes CSA. Misinterpretation commonly arises from imprecise terminology, binary reasoning, and overreliance on physical findings as proof. This review emphasizes the need for standardized, evidence-based communication in medical reports and testimony to prevent medico-legal misunderstanding. Practical language guidance is provided to assist clinicians in accurately conveying the limitations and significance of normal findings. Understanding that “normal is common” in CSA evaluations is essential to reducing harm, supporting trauma-informed care, and strengthening the integrity of multidisciplinary decision-making.

## Introduction and background

The medical evaluation of suspected child sexual abuse (CSA) sits at a uniquely sensitive intersection of pediatrics and forensic medicine. Clinically, the examination aims to ensure safety, identify injuries or infections needing treatment, document findings, and address the child’s health and psychosocial needs. Forensically, it can support evidence collection, documentation quality, and expert interpretation that may later influence investigative and legal decisions [1-8].

Despite strong professional guidance, a persistent and harmful misconception remains: if the anogenital examination is normal, abuse did not happen. This “physical proof” expectation is understandable in legal settings where physical evidence is often perceived as decisive. However, it is inconsistent with the pediatric evidence base, including legally confirmed abuse cases. The foundational message from classic pediatric work remains relevant: “it’s normal to be normal” [2,4-12].

Large clinical series consistently show that most children evaluated for suspected CSA have normal or nonspecific findings, and definitive signs of penetrating trauma are uncommon, particularly when the exam is nonacute [1,5,10,11]. In one legally confirmed series, abnormal genital findings in girls were reported in a minority of cases, and abnormal anal findings were rare, highlighting that even “proven” abuse frequently leaves no definitive physical signature [2]. These patterns are not failures of examination - they reflect biology (rapid healing), abuse characteristics (often noninjurious acts), and timing (delayed disclosure leading to delayed exams) [1,9,13-17]. While guidelines clearly state that a normal exam does not exclude abuse, there is less standardized, evidence-based guidance on how to communicate that reality - consistently, precisely, and forensically - across (i) medical documentation, (ii) multidisciplinary case review, and (iii) testimony. Variability in wording (“negative exam,” “no evidence”) can unintentionally mislead non-medical audiences [1,3,4,7,9].

This review addresses that translation gap with a structured communication framework and practical language tools designed for high-impact publication and interdisciplinary uptake [1,7,8]. In this review, we aim to explain why most CSA exams are normal and to provide a narrative synthesis that integrates pediatric examination science with forensic implications and evidence-based communication tools [1,4,7,8].

## Review

Methods

This narrative review was developed to inform both clinical and forensic audiences. A targeted literature search strategy was designed to capture (1) consensus guidelines and national protocols; (2) large clinical series describing examination findings; (3) studies on healing and follow-up value; (4) forensic evidence recovery studies; (5) sexually transmitted infection (STI) testing and interpretation literature relevant to pediatric populations; and (6) publications addressing variability in injury detection and interpretation.

Databases and sources typically used in this field include PubMed/MEDLINE, major pediatric and forensic journals, and authoritative organizational guidance. Emphasis was placed on studies with clear examination methods, photodocumentation, and/or blinded review when available, and explicit definitions of “diagnostic” versus “nonspecific” findings. Because this is a narrative review, study selection prioritized conceptual coverage and practice relevance rather than exhaustive systematic inclusion. Findings were synthesized into thematic domains and translated into an applied reporting/testimony framework.

Narrative synthesis

How Often Are CSA Examinations “Normal”?

Across multiple decades of research, the empirical pattern has remained consistent: definitive traumatic findings are uncommon in children evaluated for CSA, particularly when examinations are conducted outside the acute timeframe [1,5,10,11]. In legally confirmed cohorts, most girls demonstrate normal or nonspecific findings, with only a minority showing abnormal genital findings and even fewer presenting with abnormal anal findings [2]. These observations are critical because they directly refute the persistent assumption that confirmed abuse should routinely produce visible injury [2,12]. Large referral-based studies reinforce this pattern. In extensive clinical cohorts of children assessed for suspected sexual abuse, examinations are frequently normal or nonspecific, and the proportion of “positive” findings is influenced by definitional thresholds and contextual factors [10,11]. Contemporary analyses further demonstrate that reported rates of positive findings vary according to examiner expertise, photodocumentation practices, injury classification criteria, and whether the case is acute or nonacute, resulting in variability across jurisdictions and healthcare systems [1,9]. From a forensic standpoint, one particularly significant observation is that repeated reported penetrative events do not necessarily correlate with definitive physical findings. In a large pediatric study, most children reporting multiple penetrative events had no definitive evidence of penetration on examination, and bleeding history showed a stronger association with definitive findings than repetition alone [12]. This evidence challenges courtroom narratives that equate penetration with inevitable structural injury [12,18].

What Does “Normal” Actually Mean? Interpretation Frameworks and Categories

The term “normal examination” becomes problematic in forensic settings when it is interpreted as a binary indicator - either abuse occurred, or it did not. Pediatric and forensic guidance instead position the examination as one component within a broader clinical-forensic evaluation [3,4,6,7]. Contemporary interpretive frameworks organize findings into structured categories: normal anatomical variants; findings attributable to medical conditions unrelated to trauma; findings that may mimic abuse; findings without consensus significance; and findings that are highly suggestive of trauma, including rare residual injuries such as healed transections in specific contexts [1,4,5]. This classification system provides conceptual clarity and reduces diagnostic ambiguity. Such structured interpretation mitigates two major errors: falsely labeling normal variants as abuse (false positives) and incorrectly excluding abuse in the absence of definitive trauma (false negatives) [1,5,9]. Over time, the catalog of normal and nonspecific findings has expanded as normative anatomy and differential diagnoses have been better characterized, whereas the group of findings considered diagnostic of penetrating trauma has remained relatively limited [1,9]. This evolution reflects scientific refinement and underscores the importance of calibrated language when communicating certainty and limitation in clinical reports and testimony [1,5].

Why Injury Is Often Absent: Biology, Mechanics, and the “Healing Problem”

Low physical yield in CSA examinations is best understood through three intersecting mechanisms. First, many abusive acts do not produce detectable injury. CSA encompasses a wide range of behaviors, including nonpenetrative contact and acts involving minimal force. Even when penetration is alleged, tissue elasticity and variation in mechanics mean that visible trauma does not uniformly occur [2,9,12]. Second, anogenital tissues often heal rapidly and with limited residual signs. Healing studies form one of the strongest empirical foundations for understanding why examinations are frequently normal. Hymenal and nonhymenal injuries demonstrate variable healing trajectories, and many resolve without persistent visible changes unless deep lacerations or transections were present [15-17]. Consequently, the diagnostic window for detecting injury may be brief, and examinations performed after that interval will often be normal, even when injury initially occurred [15-17]. Third, examinations are frequently delayed relative to the alleged event. Delayed disclosure is common in CSA, and when combined with rapid healing, it substantially reduces the likelihood of observing visible trauma [2,13-17]. Thus, the absence of injury more often reflects timing than the absence of abuse. Additionally, case-control research indicates that certain anatomical or hymenal features may differ statistically between abused and nonabused populations, but these differences are neither universal nor sufficient to transform the examination into a confirmatory test [18]. This reinforces the need for standardized interpretive criteria and cautious, disciplined courtroom phrasing [1,5,9,18-21].

Timing, Urgency, and the Role of Follow-Up Examinations

Whether a suspected CSA evaluation constitutes a medical “emergency” depends on clinical stability and forensic timing. Evidence indicates that the timing of examination after an allegation influences both injury detection and forensic evidence recovery, while also intersecting with practical triage constraints [13,22,23]. Because delays are often unavoidable due to disclosure patterns or access barriers, a normal nonacute examination should be viewed as expected rather than contradictory [13]. Follow-up examinations may provide additional value in selected circumstances. Injuries may evolve, bruising may become more apparent, symptoms may develop, or expert reassessment of documentation may be warranted [14,17]. In forensic contexts, repeat examination may help clarify whether a questionable finding resolves (suggesting transient irritation) or persists in a manner consistent with prior trauma, while acknowledging that most injuries heal without residua [14-17].

Documentation Quality, Imaging, and Diagnostic Reliability

Recognition and interpretation of injuries depend heavily on documentation quality and review systems. National and professional standards emphasize photodocumentation as a core component of pediatric CSA evaluation, recommending that both injuries and normal anatomical features be recorded [7,8]. Photographs enhance clarity, support peer review, and strengthen forensic defensibility, but they complement rather than replace detailed written documentation [8]. Guideline updates also stress the importance of access to expert reviewers, particularly when findings are potentially concerning [1,5]. Diagnostic reliability can be strengthened through structured peer review, continued training, and appropriate use of imaging technology [1,4,5]. Evidence suggests that video imaging may improve diagnostic agreement relative to still images in selected contexts, including evaluation of complex findings such as transections [5,20,21]. This underscores that although most examinations are normal, robust quality systems remain essential to accurately identify and interpret the minority of diagnostic findings [1,21].

Forensic Evidence Recovery: Why Biological Evidence Is Often Absent

A normal physical examination should not be equated with the absence of forensic evidence; however, biological evidence recovery is likewise time-sensitive and frequently low-yielding in pediatric cases. Delayed disclosure, hygiene practices, limited ejaculate deposition in many cases, and timing of collection all contribute to reduced recovery rates [22-24]. Studies of pediatric forensic evidence collection demonstrate that DNA identification and trace recovery are feasible within acute timeframes but decline substantially with delay [22,23]. Implementation of specialized pediatric sexual assault nurse examiner (P-SANE) programs has been associated with measurable improvements in evidence handling and judicial process indicators in some settings [24]. Established forensic protocols emphasize structured consent or assent, trauma-informed care, meticulous documentation, and maintenance of chain-of-custody procedures [7,8].

From a medicolegal perspective, the absence of DNA or visible injury is common and non-exclusionary, whereas the presence of biological evidence can be highly informative when properly collected and interpreted [22-24].

STI Testing and Interpretation: “Low Prevalence” Does Not Mean “Low Importance”

STI findings occupy a distinctive position within CSA evaluations. Certain infections may provide strong support for sexual contact, yet interpretation in pediatric populations requires caution due to low base rates and the possibility of nonsexual transmission in defined scenarios [1,25-28]. Current guideline logic favors targeted testing rather than reflexive universal screening. Recommendations consider factors such as reported penetration, symptoms, perpetrator risk, household exposure, local prevalence, and patient or guardian request [1,3,25]. Expansion of nucleic acid amplification test (NAAT) methodologies has improved feasibility and sensitivity but also necessitates confirmatory strategies tailored to the organism and clinical context [25,28]. Although the prevalence of gonorrhea and chlamydia in pediatric CSA populations is generally low, measurable positivity has been reported in modern cohorts [1,29]. Low prevalence heightens the need for careful test selection and nuanced interpretation to avoid missed diagnoses and false-positive conclusions [27,28]. Importantly, some infections historically linked to sexual transmission may occur through nonsexual routes in children, including perinatal transmission, particularly relevant for early-life chlamydia and rare nonsexual contact transmission in specific reports [30-33]. These realities do not diminish forensic relevance; instead, they mandate contextual, timeline-sensitive interpretation and, when appropriate, multidisciplinary consultation [25-28]. Organisms such as *Gardnerella vaginalis* may correlate with sexual contact in some pediatric data, but are not uniformly probative [34]. Similarly, human papillomavirus (HPV)-related findings and anogenital warts demonstrate heterogeneity, and systematic reviews emphasize that the presence of warts alone cannot establish abuse without contextual evaluation [35,36]. Accordingly, STI results should be communicated with precision, differentiating infections strongly suggestive of sexual transmission in children from those with plausible alternative pathways, while avoiding both overreach and under-communication [1,25-28,35,36].

Anal Findings: Limited Specificity, High Consequence of Misinterpretation

Anal findings are particularly susceptible to misinterpretation because many features may represent normal variants, medical conditions such as constipation, examination technique effects, or transient irritation rather than trauma [1,5,37,38]. Large retrospective studies comparing children with and without probable anal penetration illustrate substantial overlap and emphasize the complexity of interpretation [37]. Case-control investigations further explore specific anal signs, yet findings still require careful contextualization within established interpretive frameworks [38,39].

Forensic consequences are significant in both directions. Over-interpretation risks false certainty and potential injustice, whereas under-interpretation may reinforce the misconception that normal findings equate to the absence of abuse. Standardized frameworks and disciplined communication, therefore, become ethically and legally essential [1,5,7,9,40].

The “Misconception Cascade”: How Normal Exams Become Courtroom Myths

The medicolegal system often seeks binary conclusions, whereas CSA medicine produces probabilistic interpretations. This structural mismatch fuels a misconception cascade. First, visible injury is erroneously equated with proof of abuse, and absence of injury with absence of abuse [2,9,12,41,42]. Second, imprecise clinical language, such as “negative exam” or “no evidence,” may be interpreted as exculpatory [1,3,7]. Third, base-rate neglect obscures the fact that most cases present nonacutely, when healing has already occurred, and normal findings are expected [13-17]. Fourth, assumptions that penetration inevitably produces tissue disruption are contradicted by pediatric and adolescent evidence [12,42]. Fifth, broader injury-detection variability in adult and adolescent sexual assault contexts further illustrates that injury rates depend on method, timing, and definitions rather than serving as simple proof indicators [43,44].

System-level factors amplify these misconceptions, including variability across practice settings, inconsistent documentation standards, and limited access to expert review [1,7,8,21]. Emergency department detection studies demonstrate that identification depends on systems and training as much as on physical signs [40]. Additionally, reported symptoms alone do not reliably predict diagnostic findings, underscoring the need for comprehensive evaluation rather than symptom-based inference [41].

This analysis highlights an actionable publication gap: while many sources acknowledge that normal examinations are common, fewer provide structured guidance on communicating normality without negating allegations [1,3,7,9]. Table 1 synthesizes the biological and procedural drivers of normal examinations with corresponding forensic implications and recommended reporting approaches.

**Table 1:** Why most CSA exams are normal: drivers, forensic implications, and what to communicate CSA: child sexual abuse; STI: sexually transmitted infection; DNA: deoxyribonucleic acid; NAAT: nucleic acid amplification test

Driver of low physical yield	What the evidence shows	Forensic implication	Evidence-based message to use in reports/testimony
Delayed disclosure → delayed exam	Many exams occur after the event; timing affects detection [13].	Normal exam may reflect timing, not the absence of abuse.	“The exam was performed nonacutely; absence of injury is expected because healing can occur rapidly” [13-17].
Rapid healing of anogenital injuries	Many hymenal/nonhymenal injuries resolve with minimal residua unless deep transection occurs [15-17].	“No injury” is not probative for “no assault.”	“Genital tissues may heal quickly; normal findings do not rule out abuse” [2,15-17].
Many abusive acts are noninjurious	CSA often does not produce visible trauma; even reported penetration may not correlate with definitive findings [12,42].	Injury absence cannot negate a credible history/disclosure.	“A normal exam is common in children with disclosures of abuse; the history remains central” [2,12,42].
Definitions and examiner variability	Positive finding rates vary by criteria, training, setting, and country [1,9,10].	Courts may compare cases unfairly if the criteria differ.	“Interpretation depends on standardized criteria; this report uses guideline-based categories” [1,4,5].
Documentation quality affects reliability	Photodocumentation is standard of care; video may improve agreement for complex findings [8,21].	Poor images increase diagnostic uncertainty and disputes.	“Images were obtained to permit expert review and improve reliability of interpretation” [1,8,21].
Forensic evidence recovery is time-sensitive	DNA/evidence yield is feasible acutely but commonly absent with delay [22,23].	No DNA does imply no abuse.	“Biological evidence is often absent in delayed presentations; this does not exclude abuse” [22,23].
STI prevalence is low but meaningful	STIs are uncommon overall; NAAT use requires careful interpretation; some infections have nonsexual pathways [25-33].	Over- or under-interpretation can distort legal meaning.	“STI results must be interpreted with method, timeline, and known transmission pathways; findings may support sexual contact but are not always exclusive” [25-28,30-36].
Symptoms are nonspecific	Symptoms alone may not predict diagnostic findings [41].	Symptom-based “proof” narratives are unreliable.	“Symptoms are important clinically but are not specific for CSA; interpretation requires full context” [41].

Tables 2-3 offer standardized language options and phrasing cautions to reduce medico-legal misinterpretation [1,7,8].

**Table 2:** Evidence-based documentation and testimony language for CSA evaluations CSA: child sexual abuse; STI: sexually transmitted infection; DNA: deoxyribonucleic acid

Clinical scenario	Recommended wording	Rationale	Key supporting evidence
Non-acute exam with normal findings	“The anogenital examination is normal. This finding is common in children evaluated for suspected sexual abuse and does not exclude abuse.”	Most CSA exams are normal, even in confirmed cases; normal findings are expected.	[1-3,10-12]
Explaining healing	“Genital and anal tissues may heal rapidly. Absence of visible injury at the time of examination does not indicate that injury did not occur.”	Rapid healing documented in pediatric injury studies.	[15-17]
Courtroom clarification	“A normal examination cannot confirm or rule out whether abuse occurred. It indicates that no diagnostic injury is visible today.”	Prevents binary misinterpretation (“normal = no abuse”).	[1-6]
When penetration is alleged but no findings are present	“Physical findings do not allow reliable determination of whether penetration occurred. Many children with reported penetration have normal examinations.”	Penetration does not reliably correlate with visible trauma.	[12,42]
Documentation quality statement	“Photodocumentation was obtained to enhance diagnostic accuracy and allow expert review.”	Imaging improves reliability and defensibility.	[1,7,8,21]
STI testing explanation	“STI testing was performed based on guideline indications. Positive findings require contextual interpretation including timing and transmission pathways.”	Low prevalence + need for nuanced interpretation.	[25-28,30-36]
Explaining the absence of DNA evidence	“Absence of forensic biological evidence is common, particularly when evaluation occurs after a delay.”	Evidence recovery declines with time; absence is non-exclusionary.	[22-24]
Multidisciplinary summary statement	“Medical findings should be interpreted alongside the child’s history, investigative findings, and psychosocial assessment.”	CSA assessment is multidimensional.	[3,7,42]

**Table 3:** Phrases to avoid in CSA reports and safer evidence-based alternatives CSA: child sexual abuse; STI: sexually transmitted infection

Phrase to avoid	Why it is problematic	Recommended alternative	Supporting evidence
“Negative exam”	Implies abuse did not occur	“Normal examination; no diagnostic findings observed at this time.”	[1-5]
“No evidence of abuse”	Misleading binary conclusion	“No physical findings diagnostic of trauma were identified.”	[1,3,9]
“The hymen is intact, therefore no penetration occurred.”	Scientifically incorrect; ignores healing and elasticity	“The examination does not allow reliable determination regarding penetration.”	[12,15-17,42]
“The exam proves abuse.”	Overstates medical certainty	“Certain findings may be highly suggestive of trauma; interpretation must follow guideline criteria.”	[1,4,5]
“Symptoms confirm abuse.”	Symptoms are nonspecific	“Symptoms are clinically relevant but not specific for CSA.”	[41]
“STI confirms abuse” (without context).	Some infections have non-sexual transmission routes	“Certain STIs are concerning for sexual transmission; interpretation depends on age, organism, testing method, and clinical context.”	[25-28,30-36]
“No injuries were found, so nothing happened.”	Reinforces common courtroom myth	“Absence of visible injury is common and does not exclude abuse.”	[2,12,42]
“Findings are consistent with sexual assault” (without clarification).	May be misunderstood as proof	“Findings are consistent with the history provided; however, many cases have normal examinations.”	[1,3,4]

Discussion

What Is Genuinely “New” Here?

The central contribution of this review is not the reiteration that most CSA examinations are normal - this has been consistently demonstrated across large clinical cohorts and confirmed cases [1,2,10,11]. Rather, the novelty lies in the structured translation of established clinical evidence into a coherent medico-legal communication framework. While pediatric research has clarified healing dynamics, anatomical variability, and diagnostic limitations [15-18], and forensic guidance has emphasized documentation standards and evidence handling [7,22-24], these domains are often discussed in parallel rather than integrated. This review bridges that divide by proposing a deliberate communication model that is simultaneously clinically accurate, forensically precise, and legally resistant to misinterpretation. Clinical accuracy requires alignment with empirical healing and anatomy data [15-18]. Forensic precision demands explicit articulation of what the examination can and cannot establish, particularly regarding timing and evidentiary limits [7,22-24]. Legal defensibility requires proactive correction of the pervasive “normal = no abuse” inference without overstating certainty [1-3,12,42]. In this sense, the innovation is not diagnostic but translational: treating documentation language as a structured intervention that protects both patient welfare and judicial integrity [1,3,7,8].

Practical Recommendations for High-Integrity Reporting and Testimony

High-quality CSA reporting should clearly distinguish between objective medical observations and interpretive commentary. Explicit separation of “Objective Findings” and “Interpretation/Limitations” sections anchors the report in observable facts while transparently addressing timing and healing considerations [1,5,15-17]. This structural clarity reduces ambiguity and enhances credibility in both clinical and courtroom settings. Terminology is equally critical. Replacing the phrase “negative exam” with “normal exam that is not inconsistent with the history” aligns with pediatric interpretive standards and prevents binary misreading [1-4,42]. This wording acknowledges the absence of diagnostic trauma while avoiding the implication that abuse did not occur. Explicitly stating why normal findings are common, such as rapid tissue healing or the fact that many abusive acts do not produce visible injury, further reduces misinterpretation [2,13-17]. Documentation processes should reflect contemporary standards. Photodocumentation enhances transparency, facilitates expert review, and strengthens reliability [1,7,8,21]. When findings are concerning or ambiguous, access to peer or expert review supports diagnostic consistency and defensibility [1,7,8,21]. STI results require particularly careful contextual framing. Reports should specify the organism identified, the testing methodology (including NAAT use), any confirmatory strategy employed, and the interpretive statement that addresses both forensic significance and known nonsexual transmission possibilities where relevant [25-28,30-36]. This balanced approach prevents both overreach and unwarranted minimization. These reporting principles are operationalized in Table 2 (recommended phrasing) and Table 3 (phrases to avoid and rationale) and synthesized within Table 1 to link biological mechanisms with communication strategy [1,7,8].

Research Gaps and Trends

Emerging trends suggest that CSA evaluation is evolving toward measurable quality and documentation standards. Increasing emphasis on photodocumentation, secure storage, and structured expert review reframes documentation as an auditable outcome rather than a passive record [1,7,8,21]. Future research should examine how documentation quality influences diagnostic agreement and downstream legal clarity. Technological developments also warrant systematic evaluation. Evidence that video imaging may improve interpretive agreement compared with still images suggests opportunities for standardized imaging protocols and multicenter reliability studies [5,21]. Cross-disciplinary trials assessing interrater reliability across child abuse pediatricians, forensic nurses, and general pediatric clinicians would strengthen evidence-based training models. STI testing represents another evolving frontier. As NAAT methodologies become more widely implemented in low-prevalence pediatric populations, research should focus on confirmatory testing algorithms, base-rate-informed interpretation frameworks, and risk mitigation strategies to minimize false-positive or misinterpreted results [25-28,30-36]. A particularly important gap concerns communication outcomes. Although guidelines emphasize careful phrasing, there is limited empirical research evaluating how specific medical-report language influences judicial interpretation, caregiver understanding, or child well-being. Given the documented misconception cascade, studies examining standardized language tools such as those presented in Tables 2-3 could treat communication as a measurable intervention [1,3,7,42,45]. Finally, global and population diversity gaps remain. Normative anatomical data and interpretive frameworks have historically been developed within limited geographic and demographic contexts. Expanding high-quality normative and case-control studies across diverse populations may reduce both false-positive and false-negative interpretations and enhance international consistency [1,9,18].

Limitations

This narrative review emphasizes conceptual synthesis and translational application rather than exhaustive systematic inclusion. As such, it does not provide pooled quantitative estimates or formal meta-analytic weighting. The underlying literature itself is heterogeneous, with variability in examination techniques, documentation standards, injury definitions, and criteria used to define “confirmed” abuse [1,9,10]. In many studies, the absence of a definitive gold standard for abuse confirmation further complicates interpretation. These limitations underscore the importance of cautious language and structured communication - precisely the translational emphasis advanced in this review.

Forensic Implications and Future Directions

The predominance of normal findings in CSA examinations requires a fundamental shift in forensic thinking from injury-centered reasoning to interpretation-centered practice. In many cases, the vulnerability is not a diagnostic failure but communicative imprecision. The medical report is itself a forensic document, and the way normal findings are framed can either prevent or perpetuate the misconception that absence of injury equals absence of abuse. Clear articulation of timing, healing dynamics, and evidentiary limits is therefore not optional; it is integral to justice. A novel direction for the field is to treat communication standardization as a measurable forensic intervention. Structured reporting templates and carefully calibrated language should be evaluated for their impact on judicial interpretation, interprofessional consistency, and reduction of adversarial misrepresentation. Rather than focusing exclusively on improving detection of rare diagnostic injuries, future research should examine how documentation structure, imaging protocols, and expert phrasing influence reliability and legal clarity. Additionally, expanding high-quality normative anatomical data across diverse populations will reduce interpretive uncertainty and strengthen evidentiary precision. The next phase of advancement in CSA forensic medicine lies not only in better identifying injury, but in refining how normal examinations are explained - consistently, transparently, and defensibly - within multidisciplinary and legal contexts.

## Conclusions

Most CSA examinations are normal, not because abuse is rare, but because injury is not an inevitable or durable outcome of abuse. Rapid tissue healing, variability in contact mechanics, delayed disclosure, and evidentiary timing collectively explain why visible trauma is the exception rather than the rule. The expectation of physical proof reflects a misunderstanding of pediatric biology rather than a limitation of medical evaluation. The true forensic challenge is therefore interpretive, not diagnostic. A normal examination neither confirms nor excludes abuse; it defines the boundaries of what can be medically observed at a specific moment in time.

When clinicians fail to explicitly communicate these boundaries, the vacuum is often filled by binary legal assumptions that distort medical meaning. Advancing the field requires a deliberate shift from injury-centered thinking to framework-centered interpretation. High-integrity documentation, standardized language, and disciplined explanation of limitations should be recognized as core components of forensic quality. In this context, communication is not ancillary - it is evidentiary. By reframing the “normal exam” as an expected and scientifically grounded finding, and by operationalizing how it is conveyed across clinical and legal systems, pediatric forensic practice can move toward greater clarity, consistency, and justice.

## References

[1] Kellogg ND, Farst KJ, Adams JA (2023). Interpretation of medical findings in suspected child sexual abuse: an update for 2023. Child Abuse Negl.

[2] Adams JA, Harper K, Knudson S, Revilla J (1994). Examination findings in legally confirmed child sexual abuse: it's normal to be normal. Pediatrics.

[3] Jenny C, Crawford-Jakubiak JE (2013). The evaluation of children in the primary care setting when sexual abuse is suspected. Pediatrics.

[4] Adams JA, Kellogg ND, Farst KJ (2016). Updated guidelines for the medical assessment and care of children who may have been sexually abused. J Pediatr Adolesc Gynecol.

[5] Adams JA, Farst KJ, Kellogg ND (2018). Interpretation of medical findings in suspected child sexual abuse: an update for 2018. J Pediatr Adolesc Gynecol.

[6] Adams JA, Kaplan RA, Starling SP (2007). Guidelines for medical care of children who may have been sexually abused. J Pediatr Adolesc Gynecol.

[7] (2026). U.S. Department of Justice, Office on Violence Against Women. A national protocol for sexual abuse medical forensic examinations - pediatric. https://www.justice.gov/ovw/file/846856/download.

[8] (2026). International Association of Forensic Nurses. KIDSta protocol section B6: photo-documentation. https://www.safeta.org/page/kidssectionb6/.

[9] Pillai M (2008). Genital findings in prepubertal girls: what can be concluded from an examination?. J Pediatr Adolesc Gynecol.

[10] Heger A, Ticson L, Velasquez O, Bernier R (2002). Children referred for possible sexual abuse: medical findings in 2384 children. Child Abuse Negl.

[11] Smith TD, Raman SR, Madigan S, Waldman J, Shouldice M (2018). Anogenital findings in 3569 pediatric examinations for sexual abuse/assault. J Pediatr Adolesc Gynecol.

[12] Anderst J, Kellogg N, Jung I (2009). Reports of repetitive penile-genital penetration often have no definitive evidence of penetration. Pediatrics.

[13] Watkeys JM, Price LD, Upton PM, Maddocks A (2008). The timing of medical examination following an allegation of sexual abuse: is this an emergency?. Arch Dis Child.

[14] Gavril AR, Kellogg ND, Nair P (2012). Value of follow-up examinations of children and adolescents evaluated for sexual abuse and assault. Pediatrics.

[15] McCann J, Miyamoto S, Boyle C, Rogers K (2007). Healing of hymenal injuries in prepubertal and adolescent girls: a descriptive study. Pediatrics.

[16] McCann J, Miyamoto S, Boyle C, Rogers K (2007). Healing of nonhymenal genital injuries in prepubertal and adolescent girls: a descriptive study. Pediatrics.

[17] Heppenstall-Heger A, McConnell G, Ticson L, Guerra L, Lister J, Zaragoza T (2003). Healing patterns in anogenital injuries: a longitudinal study of injuries associated with sexual abuse, accidental injuries, or genital surgery in the preadolescent child. Pediatrics.

[18] Berenson AB, Chacko MR, Wiemann CM, Mishaw CO, Friedrich WN, Grady JJ (2000). A case-control study of anatomic changes resulting from sexual abuse. Am J Obstet Gynecol.

[19] Gallion HR, Milam LJ, Littrell LL (2016). Genital findings in cases of child sexual abuse: genital vs vaginal penetration. J Pediatr Adolesc Gynecol.

[20] Garfield GB, Schou MP, Lassen K, Leth PM (2021). Hymenal transections in children found by photocolposcopy in suspected sexual abuse cases is associated with a history of bleeding. J Forensic Leg Med.

[21] Killough E, Spector L, Moffatt M, Wiebe J, Nielsen-Parker M, Anderst J (2016). Diagnostic agreement when comparing still and video imaging for the medical evaluation of child sexual abuse. Child Abuse Negl.

[22] Thackeray JD, Hornor G, Benzinger EA, Scribano PV (2011). Forensic evidence collection and DNA identification in acute child sexual assault. Pediatrics.

[23] Girardet R, Bolton K, Lahoti S (2011). Collection of forensic evidence from pediatric victims of sexual assault. Pediatrics.

[24] Hornor G, Thackeray J, Scribano P, Curran S, Benzinger E (2012). Pediatric sexual assault nurse examiner care: trace forensic evidence, ano-genital injury, and judicial outcomes. J Forensic Nurs.

[25] Workowski KA, Bachmann LH, Chan PA (2021). Sexually transmitted infections treatment guidelines, 2021. MMWR Recomm Rep.

[26] Hammerschlag MR (2011). Sexual assault and abuse of children. Clin Infect Dis.

[27] Hammerschlag MR, Guillén CD (2010). Medical and legal implications of testing for sexually transmitted infections in children. Clin Microbiol Rev.

[28] Qin X, Melvin AJ (2020). Laboratory diagnosis of sexually transmitted infections in cases of suspected child sexual abuse. J Clin Microbiol.

[29] Kellogg ND, Melville JD, Lukefahr JL, Nienow SM, Russell EL (2018). Genital and extragenital gonorrhea and chlamydia in children and adolescents evaluated for sexual abuse. Pediatr Emerg Care.

[30] Bell TA, Stamm WE, Kuo CC, Wang SP, Holmes KK, Grayston JT (1994). Risk of perinatal transmission of Chlamydia trachomatis by mode of delivery. J Infect.

[31] Schachter J, Grossman M, Sweet RL, Holt J, Jordan C, Bishop E (1986). Prospective study of perinatal transmission of Chlamydia trachomatis. JAMA.

[32] Hasui M, Kamiya T, Nakasuji K (2022). Non-sexual transmission of concurrent genital gonorrhea. Pediatr Int.

[33] Moscatelli G, Moroni S, García Bournissen F (2021). Acquired syphilis by nonsexual contact in childhood. Pediatr Infect Dis J.

[34] Ingram DL, White ST, Lyna PR, Crews KF, Schmid JE, Everett VD, Koch GG (1992). Gardnerella vaginalis infection and sexual contact in female children. Child Abuse Negl.

[35] Costa-Silva M, Fernandes I, Rodrigues AG, Lisboa C (2017). Anogenital warts in pediatric population. An Bras Dermatol.

[36] Bacopoulou F, Karakitsos P, Kottaridi C (2016). Genital HPV in children and adolescents: does sexual activity make a difference?. J Pediatr Adolesc Gynecol.

[37] Myhre AK, Adams JA, Kaufhold M, Davis JL, Suresh P, Kuelbs CL (2013). Anal findings in children with and without probable anal penetration: a retrospective study of 1115 children referred for suspected sexual abuse. Child Abuse Negl.

[38] Hobbs CJ, Wright CM (2014). Anal signs of child sexual abuse: a case-control study. BMC Pediatr.

[39] Bruni M (2003). Anal findings in sexual abuse of children (a descriptive study). J Forensic Sci.

[40] Bravo-Queipo-de-Llano B, Alonso-Sepúlveda M, Ruiz-Domínguez JA, Molina-Gutiérrez MÁ, de Ceano-Vivas La Calle M, Bueno-Barriocanal M (2022). Child sexual abuse detection in the pediatric emergency room. Child Abuse Negl.

[41] Kelly P, Koh J, Thompson JM (2006). Diagnostic findings in alleged sexual abuse: symptoms have no predictive value. J Paediatr Child Health.

[42] Kellogg ND, Menard SW, Santos A (2004). Genital anatomy in pregnant adolescents: "normal" does not mean "nothing happened". Pediatrics.

[43] Laitinen FA, Grundmann O, Ernst EJ (2013). Factors that influence the variability in findings of anogenital injury in adolescent/adult sexual assault victims: a review of the forensic literature. Am J Forensic Med Pathol.

[44] Lincoln C, Perera R, Jacobs I, Ward A (2013). Macroscopically detected female genital injury after consensual and non-consensual vaginal penetration: a prospective comparison study. J Forensic Leg Med.

[45] Modelli ME, Galvão MF, Pratesi R (2012). Child sexual abuse. Forensic Sci Int.

